# Two-photon lithography for customized microstructured surfaces and their influence on wettability and bacterial load

**DOI:** 10.1186/s41205-024-00211-4

**Published:** 2024-04-17

**Authors:** Sophie Nilsson Zagiczek, Matthias Weiss-Tessbach, Manuel Kussmann, Doris Moser, Martin Stoiber, Francesco Moscato, Heinrich Schima, Christian Grasl

**Affiliations:** 1 Center for Medical Physics and Biomedical Engineering, Medical University of Vienna, Waehringer Guertel 18-20, AKH 4L, 1090 Vienna, Austria; 2 Department of Medicine I, Division of Infectious Diseases and Tropical Medicine, Medical University of Vienna, 1090 Vienna, Austria; 3 Department of Cranio-Maxillofacial and Oral Surgery, Medical University of Vienna, 1090 Vienna, Austria; 4 Ludwig Boltzmann Institute for Cardiovascular Research, 1090 Vienna, Austria; 5 Austrian Cluster for Tissue Regeneration, 1090 Vienna, Vienna, Austria; 6 Department for Cardiac Surgery, Medical University of Vienna, 1090 Vienna, Austria

## Abstract

**Background:**

Device-related bacterial infections account for a large proportion of hospital-acquired infections. The ability of bacteria to form a biofilm as a protective shield usually makes treatment impossible without removal of the implant. Topographic surfaces have attracted considerable attention in studies seeking antibacterial properties without the need for additional antimicrobial substances. As there are still no valid rules for the design of antibacterial microstructured surfaces, a fast, reproducible production technique with good resolution is required to produce test surfaces and to examine their effectiveness with regard to their antibacterial properties.

**Methods:**

In this work various surfaces, flat and with microcylinders in different dimensions (flat, 1, 3 and 9 μm) with a surface area of 7 × 7 mm were fabricated with a nanoprinter using two-photon lithography and evaluated for their antibiofilm effect. The microstructured surfaces were cultured for 24 h with different strains of *Pseudomonas aeruginosa* and *Staphylococcus aureus* to study bacterial attachment to the patterned surfaces. In addition, surface wettability was measured by a static contact angle measurement.

**Results:**

Contact angles increased with cylinder size and thus hydrophobicity. Despite the difference in wettability, Staphylococcus aureus was not affected by the microstructures, while for Pseudomonas aeruginosa the bacterial load increased with the size of the cylinders, and compared to a flat surface, a reduction in bacteria was observed for one strain on the smallest cylinders.

**Conclusions:**

Two-photon lithography allowed rapid and flexible production of microcylinders of different sizes, which affected surface wettability and bacterial load, however, depending on bacterial type and strain.

## Background

Antibacterial surfaces are highly requested in preventing pathogens from adhering to surfaces of various medical implants. A bacterial infection associated with any kind of implant is often related with severe complications for the patient, such as chronic relapsing disease and implant replacement is often required in this case [1].

As a survival mechanism of bacteria, they attach to the surface and form a biofilm. This condition was first observed by Van Leeuwenhoek on his own tooth in 1676 [2]. However, it did not become of interest to the medical community until the 1970s, when Nils Høiby recognized a link between infections and bacterial colonies in cystic fibrosis patients [3]. Biofilms can appear on surfaces such as living tissues, implants and other medical devices and often lead to serious problems [4]. Especially in the medical field, biofilms are a problem for human health, e.g., infections related to dental caries, infective endocarditis and cystic fibrosis pneumonia [5, 6]. Implantable medical devices are also affected by biofilms, as the solid-liquid interface on the medical devices provides an ideal environment for bacterial attachment and growth. A major problem associated with biofilms is that once the bacteria have colonized and formed a biofilm, they are irreversible and cannot be eliminated by gentle rinsing [4]. Two bacteria commonly associated with implantable medical device infections are: *Pseudomonas aeruginosa (P. aeruginosa)* and *Staphylococcus aureus (S. aureus)* [1]. They are also capable of forming biofilms, meaning that infections are often hardly treated [7, 8]. Due to the rising problem with antibiotic resistant bacteria and also the major problem of treating device-related infections, researchers have made several approaches on how to reduce the high rates of infections by including use of various materials, antibiotic coatings, attachment of covalent antimicrobial molecules and in the recent years, adjustment to the surface topography [9–12]. To date, numerous studies have been conducted in the medical field to particular test the effect of microstructures on bacterial adhesion [13, 14]. However, these studies include not only adaptations of surface structures but also involve factors such as chemical treatments [15].

So far, inspiration has come primarily from naturally occurring antibacterial surfaces, whose hierarchical structures alter the wetting of the surface and thus generate antibacterial properties. The wetting of surface structures is probably one key parameter for antibacterial surfaces but due to the many topographical parameter hardly to predict. In recent approaches also artificial intelligence was used to predict wettability of surface patterns [16, 17]. For example, a hydrophobic surface with a larger contact angle most likely will prevent bacterial adhesion, whereby smaller contact angles will increase the bacterial attachment [11]. Specifically, surfaces inspired by sharkskin have proven outstanding results for inhibiting the bacterial growth, but also inhibition of the biofilm formation for *P. aeruginosa* and *S. aureus* [18].

However, as there is no consensus in the literature for the optimal topographical features to prevent bacterial adhesion and biofilm formation, a fast, reproducible production technique with sufficient resolution is required to produce test surfaces and to examine their effectiveness with regard to their antibacterial properties [11, 19].

Fabricating such challenging three-dimensional biomedical microstructures is very complex [14, 20]. This has driven the development of various micro/nanofabrication technologies such as deep UV lithography, electron beam lithography, and Ink-jet printing. However, slow production time, limited resolution and high cost are some of the problems associated with these technologies [21]. In the early 1980s, lasers were developed that could emit ultra-short pulses of light, so-called femtosecond lasers [22]. It was found that only a short interaction time was required to produce a high-power density in the exposed material, which in turn leads to rapid photon energy transfer. Combined with further findings in the 2000s, this led to the development of two-photon polymerization (TPP) [23]. The usage of TPP was first demonstrated by Maruo et al. in 1997 [24]. In recent years, TPP has proven to be an excellent suitable method for well-defined 3D structures [25]. In this process, two-photon absorption is exploited, and the structures are fabricated layer by layer using a pulsed infrared (IR) laser [23]. The focused laser triggers a photochemical reaction in a photosensitive resin. Within the region of the focused laser, called a voxel, the material absorbs two photons in the near IR spectrum with the energy exceeding a necessary threshold leading to polymerization in this volume [26].

In this work we tested TPP for the fast and flexible fabrication of arbitrary three-dimensional microstructures to simplify and accelerate the search for effective antibacterial surfaces. Microcylinders, with only one changeable aspect of geometry, were fabricated and tested for their wettability and bacterial load, without any additional adjustments, such as changes in the material or treatments of the surface.

## Methods

### Fabrication of the surface patterns

**Fig. 1:**
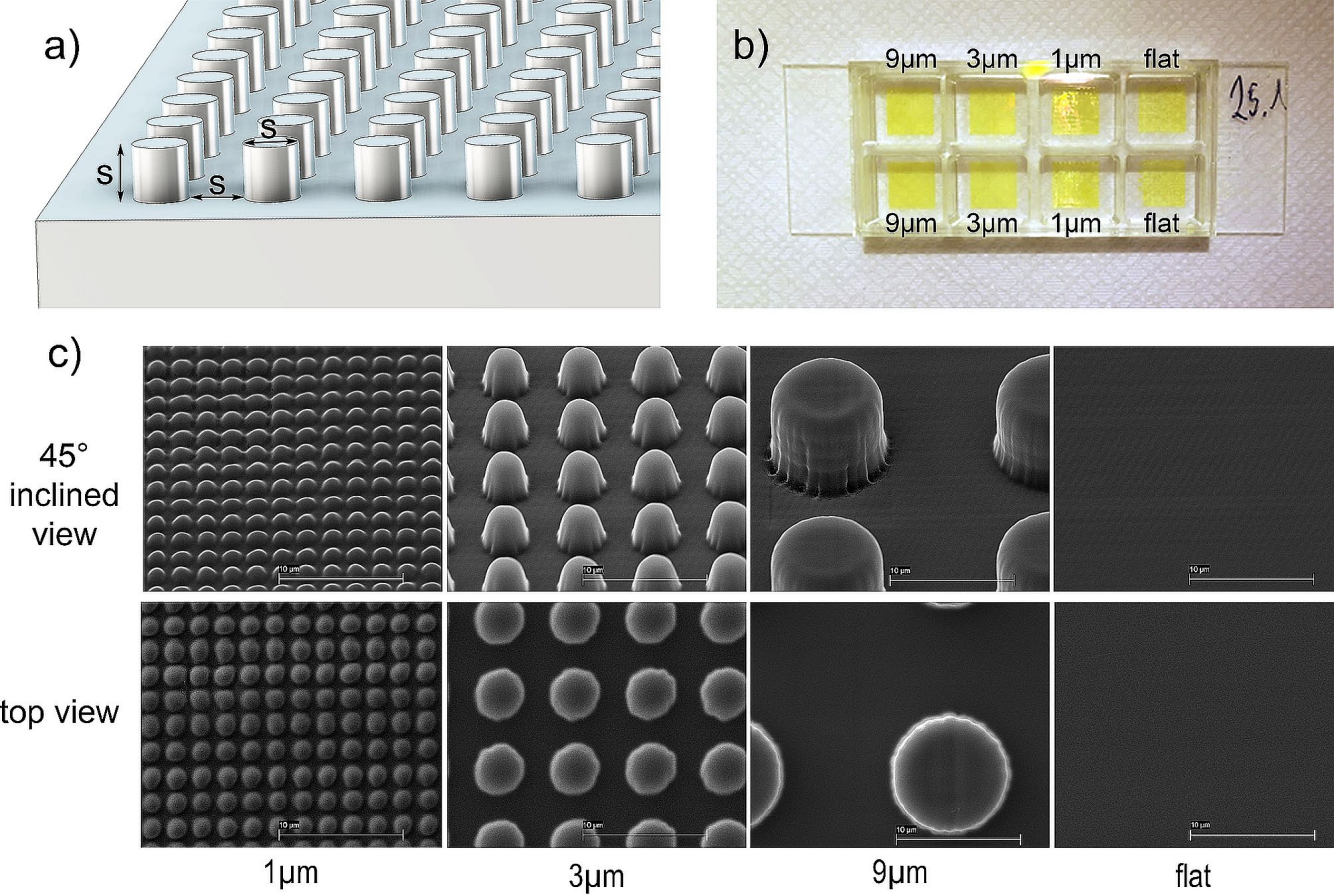
**(a)** Depiction of the designed microcylinders. The cylinders protruded from a flat base plate. The dimensional parameter s defined the diameter, the height and the distance between the cylinders. s = 1, 3 and 9 μm; **(b)** Photograph and arrangement of the of the printed structures in an 8well µ-Slide for the further bacterial assay; **(c)** Scanning electron micrographs of the printed microcylinders from an 45° inclined view and from the top. All images were taken with a magnification of 8000. Scale bar = 10 μm

Five different surfaces were compared to investigate wettability and the bacterial response. The studied microstructures consisted of cylinders emerging from a flat surface of 60 μm in height. The size and position of the cylinders was described with a size constant “s”. The diameter, the height as well as the distance between the cylinders was defined as s = 1, 3 and 9 μm (Fig. 1a).

The fourth surface type was flat and printed from the same material as the three microstructures. The 5th surface type was a flat glass control.

The surfaces were produced with a high-resolution nanoprinter (NanoOne 1000, UpNano GmbH, Vienna, Austria) that uses two-photon lithography. For the investigation of antibacterial properties, the surfaces with an area of 7 × 7 mm were printed directly in 8 well µ-Slide cell culture dishes (Fig. 1b) with a glass bottom from ibidi (ibidi GmbH, Gräfelfing, Germany). To achieve good adhesion of the printed structures in the wells, they were silanized beforehand. Surfaces were printed with a 20x objective (UPLSAPO20X, 20x/0.85 oil, Olympus Corporation, Tokyo, Japan) and the polymeric material UpBrix (UpNano GmbH, Vienna, Austria) was used. To print in the wells of the cell culture dishes, the bottom-up mode was used. There, the laser beam is focused through the high-precision glass bottom of the chambered coverslip and the structure is built from bottom to top.

The printed surface structures in the cell culture dishes with their high walls were not suitable for determining the contact angle. Therefore, the same structures were printed on glass substrates (10 × 10 × 2.66 mm) in VAT mode. In this process, a vat with a precision glass window is located above the lens during printing, preventing the resin from coming into contact with the lens and maintaining the focusing power of the lenses. During printing, the model adheres to the glass substrate and is drawn up out of the material vat. In this mode objects with a height of up to 40 mm are producible.

With the used slicing software Think3D® (UpNano GmbH, Vienna, Austria) all models were printed in adaptive mode. Thereby, the software distinguishes between low-resolution and high-resolution areas and adapts the laser voxel size accordingly. The flat basement structure was printed with an enlarged focal point and thus faster, while the fine details of the cylinders were structured with a smaller more precise focal point. For this study, the objects were sliced in conservative mode to ensure that all cylinders were printed, meaning that the focus point (voxel) is printed as soon as an element of the cad object is visible within the voxel. The printing speed for the flat basement structure was 300 mm/s and for the cylinders set to 200 mm/s. The hatching distance for the flat basement structure was in x/y = 2.5 μm and for z = 2 μm. The cylinders were structured with x/y = 0.2 μm and in z = 0.8 μm. Further, an overlap of 8 μm and a block offset of 50% was used to strengthen the connection between the fields printed separately.

After printing, the objects were cleaned with isopropanol and air-dried. The cell culture dishes with the surfaces for the bacterial studies were sterilized with ethylene oxide.

### Contact angle measurement

The wettability of the surfaces under investigation was determined by means of the sessile drop method. A drop shape analyzer DSA25E (Krüss GmbH, Hamburg, Germany) with an automatic pressure-based dosing unit (Liquid needle system, Krüss GmbH, Hamburg, Germany) and the software Advance® (Krüss GmbH, Hamburg, Germany) were used to automatically measure the contact angle with deionized water at room temperature. All surfaces were measured by portioning a drop with a volume of 0.5 µL and 1 µL ten times. A glass control was included as a control measurement to determine the differences between a flat surface of UpBrix® and a smooth glass surface. All contact angle measurements were carried out in a class 1000 clean room three seconds after deposition of the water drop under climatic clean room conditions of 25 °C and 35% relative humidity.

### Bacterial assay

The tested bacteria (*P. aeruginosa* and *S. aureus*) were obtained either from American Type Culture Collection (ATCC) or routinely obtained blood cultures of the Vienna General Hospital. One MRSA strain (ATCC 33592) was included for reasons of interest, and the others were selected based on their ability to form biofilms. All isolates were frozen and stored at − 80 °C, and before usage, pre-cultured on BD BBLTM Columbia agar plates containing 5% sheep blood (COS plates, BDTM, New Jersey, U.S.) for 24 h at 37 °C and 40% humidity. To avoid unwanted contamination, precultivation and all dilutions were performed under a biological safety level II cabinet. Colonies from a subcultured agar were dissolved in 0.9% NaCl until a McFarland of 1 for *P. aeruginosa* and 0.5 for *S. aureus* was reached. The bacterial suspension was diluted 1:10 in fresh brain-heart infusion broth and mixed. 200 µL of the diluted bacterial suspension were added to each well of the printed 8-well ibidi plates (ibidi GmbH, Gräfelfing, Germany) and incubated for 24 h at 37 °C and 40% humidity. All surfaces were tested in triplicates with the same bacterial suspension from each strain. After incubation on the structured surface, the medium was discarded, and the plates were washed with 0.9% NaCl three times. Afterwards, 0.9% NaCl were added to the wells and the bottom was gently scratched with a pipette for several times to solve the attached bacteria. The bacterial suspensions were diluted 1:10 in 0.9% NaCl on a 96-well plate and further serially diluted at 1:10 until a final concentration of 1:10^8^ was reached. 50 µL of all dilutions were pipetted onto COS plates and incubated for 18–24 h at 37 °C and 40% humidity. After incubation, the number of visual bacterial colonies were counted, and reported as CFU/mL. A separate medium control plate was carried throughout the study.

### SEM analysis

To investigate the printed surfaces and dimensions of the cylinders, the samples were first sputtered with gold using a sputter coater Q150R ES (Quorum Technologies Ltd, United Kingdom) and afterwards imaged by scanning electron microscopy at an acceleration voltage of 10 kV (Zeiss EVO MA10, Oberkochen, Germany). The diameter (s) and the distance between the cylinder centers (2s) were measured from the top view at six points on scanning electron micrographs (x8000) with the SmartTiff V03.00.02 software (Carl Zeiss Microscopy Limited).

For SEM analysis of the bacteria on the surface structures, samples were carefully washed once with PBS, fixed in Karnovsky’s fixative (2% paraformaldehyde, 2.5% glutaraldehyde in 0.1 M phosphate puffer pH 7.4; Morphisto®, Frankfurt am Main, Germany) and dehydrated in a graded ethanol series. Ethanol dehydration was followed by chemical drying. Specimens were immersed in hexamethyldisilazane (HMDS, Sigma-Aldrich) for 5 min and air dried. After complete evaporation of HMDS, samples were fixed to specimens mounts with double-faced adhesive carbon tape, gold sputtered (Sputter Coater ACE200, Leica Microsystems, Germany) and then examined in a scanning electron microscope (JSM 6310, Jeol Ltd.®, Japan) at an acceleration voltage of 10 kV.

### Statistical analysis

The statistical analysis was performed using IBM® SPSS® v27 software (IBM corporations Inc., New York, U.S.) Contact angles and number of bacteria were expressed as average ± standard deviation. Multiple group comparison of variances was evaluated using one-way ANOVA test. Bonferroni’s post hoc test was carried out to determine statistical significance.

## Results

### Printed surface patterns

Figure 1b shows a well plate with the pattern layout. One of the structures with an area of 7 × 7 mm in a well, required a printing time of about 4 h, the flat control areas were printed in 15 min each. The printed surface structures are shown in Fig. 1c. The scanning electron micrographs show a x8000 magnification of the 1, 3, and 9 μm surface structures tested, as well as a printed flat control surface. The 45° views show that the idealized structures could not be printed completely accurately. It became apparent that there was rounding on the shaft and the top of the cylinder. The s = 1 μm structures showed a mean diameter of 1.26 ± 0.03 μm, the 3 μm structures were 3.28 ± 0.03 μm and the 9 μm structures 9.01 ± 0.03 μm. The mean distance between two adjacent cylinder centers (2s) in the s = 1 μm structures was 2.01 ± 0.05 μm, for the 3 μm structures 6.01 ± 0.02 μm and for the 9 μm structures 18. 02 ± 0.06 μm.

### Wettability

**Fig. 2:**
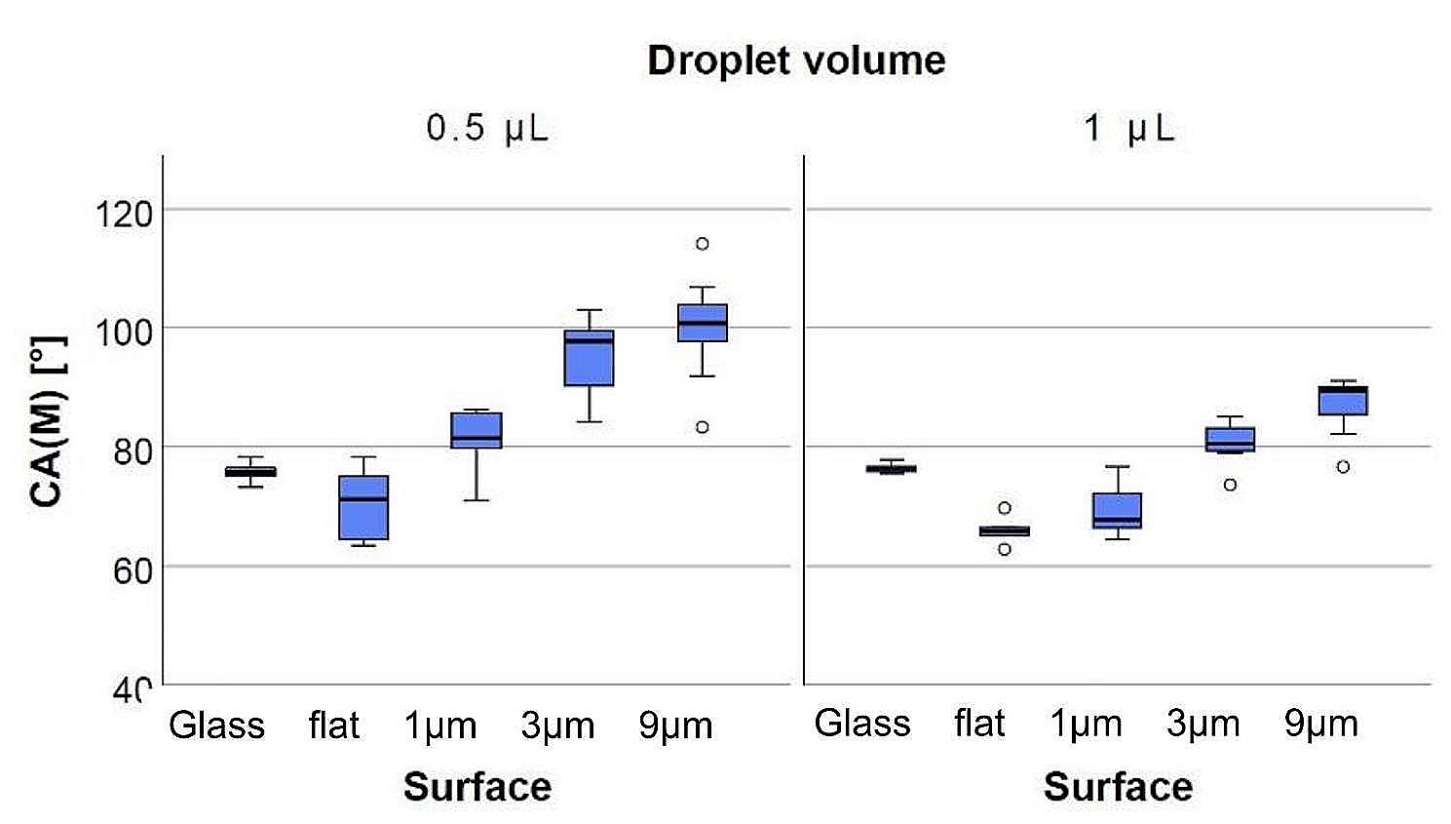
Graphic illustration of the measured contact angles of different surfaces and two droplet volumes. The contact angle increases with the increasing size of the cylinders. With larger droplet volume, the gradient rise of the contact angles was not as large as for smaller droplets

The wettability measurements showed that the flat printed surfaces were hydrophilic and that there was no significant difference (*p* = 0.193) in the contact angle due to the different drop volumes dosed. For the flat surfaces, the mean contact angle was 70.1 ± 7.9° at 0.5 µL dosing volume while for 1µL droplet volume, the mean contact angle was 65.9 ± 2.1°. In contrast, the ANOVA test revealed that there were significant differences between the different volumes on the structured surfaces (*p* = 0.003). As shown in Fig. 2, the contact angles increased with the size of the cylinders, indicating a poorer wetting. For 0.5 µL, a contact angle of 81.3 ± 5.0° for s = 1 μm cylinders, 95.0 ± 6.9° for s = 3 μm cylinders and 100.2 ± 8.0° for s = 9 μm cylinders was measured. Obviously, the wettability of the surface turns from hydrophilic to hydrophobic for this volume. For the larger droplet volume, the contact angles were slightly smaller: 69.4 ± 4.4° for s = 1 μm, 80.6 ± 3.5° for s = 3 μm and 86.9 ± 5.4° for s = 9 μm, meaning that all surfaces were hydrophilic, however reaching for hydrophobicity. The statistical analysis revealed that there were significant differences between the flat and all structured surfaces for 0.5 µL with *p*-values < 0.001 and between the flat and the larger structured surfaces (3 and 9 μm) (*p* < 0.001) for 1 µL.

### Bacterial response

**Fig. 3:**
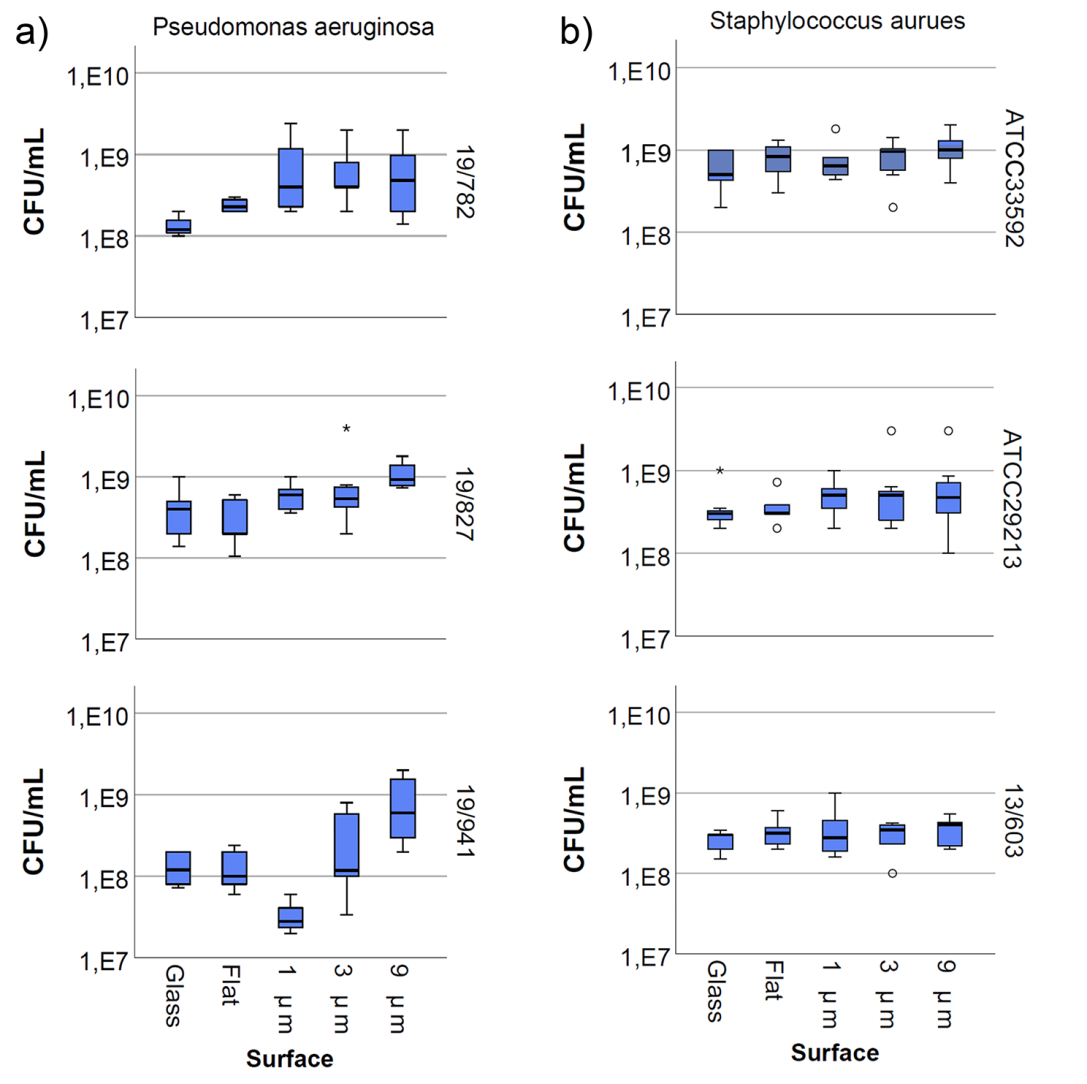
Graphic representation of colony-forming units per milliliter (CFU/mL) from the bacterial germ count after 24 h of cultivating the bacteria on different structured, flat and a neutral control surface of glass. No statistically significant differences were found between the different surfaces for any of the bacteria. However, for strain 19/827 and 19/941 the boxplot graphic indicates that the bacterial colonies increase slightly with the increasing size of the cylinders

The results showed that the used *P. aeruginosa* and *S. aureus* strains were affected differently. Figure 3 reveals that the bacterial load of strain 19/827 increased slightly with the size of the cylinders, with a maximum for the surface with 9 μm cylinders of 1.2 * 10^9^ ± 4.2 * 10^8^ CFU/mL and 6.1 * 10^8^± 2.3 * 10^8^ CFU/mL for the 1 μm structure. Nevertheless, statistical analysis did not confirm significant differences (*p* = 0.240). A similar increase was also seen for 19/941 (Fig. 3a). Interestingly, the bacterial count of 19/827 was lowest on the flat surface. In contrast, the bacterial count of strain 19/941 was almost ten times lower on the 1 μm structure than on the flat surface. In addition, a maximum level of bacteria on the largest structures was also observed for this strain (9.5 * 10^8^ ± 7.9 * 10^8^ CFU/mL). The ANOVA test showed that there was a significant difference between the different surfaces for this strain (*p* = 0.016). However, upon closer examination of this result, the post-hoc Bonferroni test showed no significant differences between the single groups. In contrast, 19/782 showed no increase or difference in bacterial levels. In addition, there were no significant differences in the bacterial quantity on the flat and glass control.

**Fig. 4:**
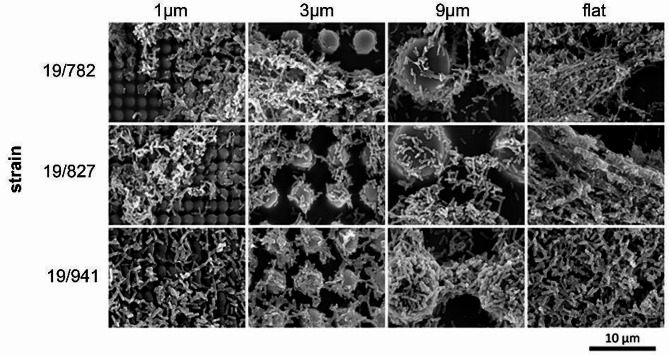
SEM images of the Pseudomonas aeruginosa adhered and cultivated on the structured surfaces. The columns represent the structure size and in the rows the tested strains are shown. All images were taken with a magnification of 2000

Figure 4 shows an overview of the scanning electron micrographs of the tested *Pseudomonas aeruginosa* strains on the different surfaces.

**Fig. 5:**
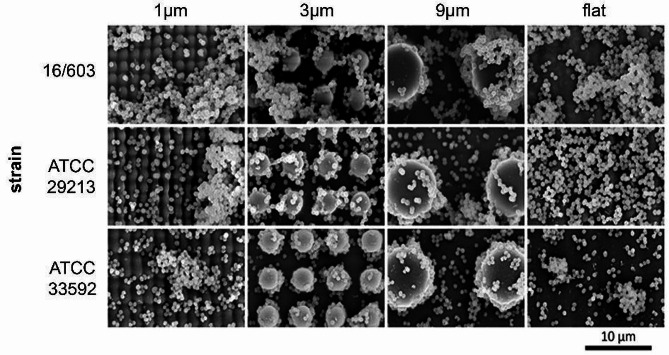
SEM images of Staphylococcus aureus adhered and cultivated on the structured surfaces. The columns represent the structure size and in the rows the tested strains are shown. All images were taken with a magnification of 2000

The experimental results of the bacterial count assay for *S. aureus* are depicted in Fig. 3b. For all strains, no significant differences (*p* = 0.635 for ATCC29213, *p* = 0.088 for ATCC 33592 and *p* = 0.496 for 13/603) in bacterial counts were observed between the different surfaces. Figure 5 shows the colonization of different surfaces with the tested *S. aureus* strains.

## Discussion

One of the key advantages of TPP is its ability to fabricate structures with sub-micron resolution, which is not possible with many other fabrication techniques. This high resolution allows for the production of intricate geometries with fine details, making it an ideal technique for applications in biomedical engineering. Unlike other techniques, such as photolithography, which are limited to planar geometries, TPP can produce arbitrary 3D structures, including overhangs and cavities. TPP is also a direct-write technique, which means that it does not require masks or templates, making it a versatile and cost-effective method for producing customized structures on demand. As there are still no valid rules for the design of antibacterial surface structures, a fast, reproducible and high-resolution production technique such as the TPP used here is required to produce test surfaces and to examine their effectiveness in terms of their antibacterial properties. The printing time of such surfaces depends on the selected objective lense that determines the resolution. The 1 μm structures represent the resolution limit with the x20 objective lens chosen in this work. (Fig. 1c). The x40 and x60 objective lenses, also available, can produce finer details, but this comes at the cost of print time due to the smaller field of view [27]. Due to the used “conservative” slicing mode, the printed features can be larger than intended in the STL file. This was particularly evident in the 1 and 3 μm structures, where the cylinders itself were printed larger, thus reducing the distance between the cylinders. Therefore, in the future, it is important to use the best possible settings in the slicer to achieve a geometrically optimized result. However, the distance between the cylinder centers corresponded exactly to the specified values in all the tested microstructures. Due to the printing process, the focal point of the laser beam in the TPP has an ellipsoidal shape with an aspect ratio between the major and minor axes of 1.5 to 3.5, depending on the numerical aperture of the laser focusing objective [28]. This results in a resolution in the z axis that is 1.5 to 3.5 times lower than in the XY plane. This may also be the reason for the rounded cylinder surfaces, which are most noticeable in the smallest structures.

For a later large-scale application of suitable antibacterial surfaces on medical implants, technologies must be selected to transfer them. This could be, for example, soft UV nanoimprint lithography, a comparatively simple and inexpensive structuring process that can be used to replicate nanostructures over large areas [29]. 

Other 3D printing processes rely on chemical strategies to achieve an antibacterial effect, such as fused deposition modelling, which uses filaments with metallic or other fillers to achieve an antibacterial effect [30]. Senderovich et al. investigated the influence of different 3D printing materials, compared them with traditionally manufactured, commercially available laryngectomy tubes and showed varying levels of biofilm colonization [12]. 

Here, a base plate with a height of 60 μm was printed under the actual structures in order to compensate for unevenness in the positioning of the well plates and to ensure the printing of the complete actual surfaces. By reducing the unevenness, this base plate could be made lower and thus the printing time could be significantly reduced again.

In this study, the wettability measurement revealed that the flat surface was slightly hydrophilic, displaying a contact angle < 90°. In addition, it was found that all surfaces were hydrophilic for larger droplet volumes and partially hydrophobic for smaller droplet volumes. Wenzel et al. noted in their work that contact angles below 90° of a smooth surface, lead to even smaller contact angles when roughness is added to the surface [31]. In contrast, larger contact angles (> 90°) on flat surfaces result in even larger contact angles when the surface becomes rougher. In this study, this relationship could not be confirmed because a contact angle < 90° was measured on the flat surface, and regardless of droplet volume, the additional microcylinders resulted in an increase in roughness that increased the contact angle for all surfaces.

The observed differences of the contact angles on the flat and structured surfaces indicated that microstructured surfaces definitely changes the hydrophobicity of the surface. This has also been confirmed by previous studies, where a water contact angle of 73 ± 3° increased to 166 ± 4° by adding nano and microstructures to the surface [32]. In 2021, Wang et al. found that not only the surface structure affected the wettability, but also the arrangement of the structures can influence the wettability differently [33]. They concluded that honeycomb arrangements produced the largest contact angles. In this study, the cylinders were arranged as a basic square with equal spacing in the horizontal and vertical direction. Rearrangement of the cylinders could induce different results and particularly the arrangement in hexagonal units would be interesting for further research.

Theoretically, the contact area between liquid and surface reduces with larger contact angles. If the liquid is contaminated with bacteria, this indeed should lead to less bacteria adhering [34]. However, in a review made by Yang (2022) [35], it was reported that several studies had determined that bacteria preferentially adhere to hydrophobic surfaces due to higher adhesion force between bacteria and surface. Nevertheless, some studies also came to the exact opposite conclusion, namely that hydrophilic surfaces tend to promote bacterial adhesion [35]. Zhang et al. (2018) found that hydrophilic and hydrophobic surfaces affect properties of *P. aeruginosa* differently. The hydrophobic surface seems to encourage extracellular polymeric substance production to form a biofilm, while the hydrophilic surface promoted the formation of strong microcolonies [36]. However, other surface properties, such as the material itself, surface patterning and hydrodynamic forces, can affect the adhesion of bacteria [35].

Bacterial attachment is described by complex processes involving detection, approaching and sensing of the surrounded surface. Although the last decades have led to an increasing knowledge of these processes, the attachment process is not yet fully understood. To date, topographical adjustments have been shown to impact various bacterial mechanisms, such as adhesiveness and biofilm formation [15]. In agreement with this statement, this study also confirmed this, as the bacterial load clearly differed depending on the surface structure. It was concluded that the surface structure alone had an impact on the bacterial adhesion, as the presented results showed that there were no differences of bacteria amount on the flat and glass control (Fig. 3). The results also showed obvious differences between the different strains, especially looking at *P. aeruginosa.* The textured surfaces had no effect on 19/782, while 19/827 and 19/941 were affected, indicating the importance of including various strains due to different bacterial characteristics. *S. aureus*, however, was much less influenced by the structured surfaces, regardless of the strain. This finding could be explained by the morphological properties and bacterial size, as this bacterium is of a smaller size than *P. aeruginosa* [37, 38]. Also, the fact that *S. aureus* is a Gram-positive (lack of outer lipid membrane and a thick peptidoglycan layer) bacteria and *P. aeruginosa* a Gram-negative (thin peptidoglycan layer with an outer lipid membrane) may affect the bacterial sensing differently. This was also confirmed by previous studies, where findings concluded that the outer membrane and cell walls may affect the bacterial sensing of the surface topography leading to different attachment behaviors [39]. However, compared to this study, the opposite was found: The Gram-negative bacterium *P. aeruginosa* had greater difficulty adhering and growing on textured surfaces than the Gram-positive bacterium *S. aureus.* Generally, existing studies include only one strain of the bacteria, and therefore the results of previous studies examining the same bacteria may differ from those of this study. In a study by Yang et al. (2015), honeycombs of the same length scale as in this study were fabricated and cultured with *S. aureus*. The results showed that pores of 1 μm size significantly reduced bacterial growth. However, larger pores of 3 and 5 μm increased the bacterial growth [40]. Nevertheless, these surface structure may influence the bacteria differently than those in this study, especially regarding the arrangement similar to honeycombs. A further study is the one from Ge et al. (2015), where micro sized pillars and their impact on bacterial behavior were investigated. In contrast to our results, they observed that *S. aureus* had difficulty adhering to pillars as small as 1 μm [41]. However, the larger pillars (10 μm and 5 μm) appeared to favor bacterial adhesion and growth, concluding that better and more attachment capabilities are present with larger structures.

Obvious differences between the surfaces occurred only for strain 19/941. The only surface on which fewer bacteria were present was the one with 1 μm cylinders. However, even if there are fewer bacteria present on this surface after 24 h of cultivation, there is a possibility that these differences will not be visible after a longer period of time. Once a bacterium has adhered and formed a biofilm, the biofilm naturally matures and disperses bacteria adhering to areas not yet contaminated. Although the result could not be confirmed statistically, the other strains indicated a trend that the bacterial load increases with the size of the cylinders. As mentioned earlier, the results from *S. aureus* demonstrated less or no difference between the surfaces, likely due to the smaller size of *S. aureus*. However, based on this, the larger structures should favor the attachment of *S. aureus*, as more attachment opportunities are available to the small bacteria, but this was not the case. Since the contact angles clearly vary on the different surfaces, it may affect other properties of the bacteria, such as biofilm formation rather than bacterial abundance.

## Conclusion

Bacterial contamination is a serious problem in the medical field. The increasing problems with antibiotic-resistant bacteria and the rising number of implant failures, contribute to the importance of conducting research on anti-adhesion surfaces. The here used method of two-photon polymerization offered a fast and flexible production of microstructures from 1 to 9 μm. The results of this study demonstrated that the size of topographic features has a significant impact on the surface wettability. Larger cylinders resulted in larger contact angles, which means that wetting is inhibited. Furthermore, the results showed that the additional micro sized cylinders on the surface also affects the behaviour of bacteria in terms of bacterial load differently, depending on the type and strain of bacteria.

## Data Availability

The datasets used and/or analyzed during the current study are available from the corresponding author on reasonable request.
